# First Eigenmode Transmission by High Efficient CSI Estimation for Multiuser Massive MIMO Using Millimeter Wave Bands

**DOI:** 10.3390/s16071051

**Published:** 2016-07-08

**Authors:** Kazuki Maruta, Tatsuhiko Iwakuni, Atsushi Ohta, Takuto Arai, Yushi Shirato, Satoshi Kurosaki, Masataka Iizuka

**Affiliations:** NTT Access Network Service Systems Laboratories, Nippon Telegraph and Telephone Corporation, 1-1 Hikarino-oka, Yokosuka-shi, Kanagawa 239-0847, Japan; iwakuni.tatsuhiko@lab.ntt.co.jp (T.I.); ohta.atsushi@lab.ntt.co.jp (A.O.); arai.takuto@lab.ntt.co.jp (T.A.); shirato.yushi@lab.ntt.co.jp (Y.S.); kurosaki.satoshi@lab.ntt.co.jp (S.K.); iizuka.masataka@lab.ntt.co.jp (M.I.)

**Keywords:** massive MIMO, multiuser MIMO, first eigenmode, millimeter wave, channel estimation, channel time variation

## Abstract

Drastic improvements in transmission rate and system capacity are required towards 5th generation mobile communications (5G). One promising approach, utilizing the millimeter wave band for its rich spectrum resources, suffers area coverage shortfalls due to its large propagation loss. Fortunately, massive multiple-input multiple-output (MIMO) can offset this shortfall as well as offer high order spatial multiplexing gain. Multiuser MIMO is also effective in further enhancing system capacity by multiplexing spatially de-correlated users. However, the transmission performance of multiuser MIMO is strongly degraded by channel time variation, which causes inter-user interference since null steering must be performed at the transmitter. This paper first addresses the effectiveness of multiuser massive MIMO transmission that exploits the first eigenmode for each user. In Line-of-Sight (LoS) dominant channel environments, the first eigenmode is chiefly formed by the LoS component, which is highly correlated with user movement. Therefore, the first eigenmode provided by a large antenna array can improve the robustness against the channel time variation. In addition, we propose a simplified beamforming scheme based on high efficient channel state information (CSI) estimation that extracts the LoS component. We also show that this approximate beamforming can achieve throughput performance comparable to that of the rigorous first eigenmode transmission. Our proposed multiuser massive MIMO scheme can open the door for practical millimeter wave communication with enhanced system capacity.

## 1. Introduction

The rapid diffusion of smartphones has triggered extensive diversification in mobile services. Not only is data traffic exploding, but also terminals are crowding public sites such as stations, airports and event venues. Unfortunately, existing frequency resources are depleted, especially in the microwave band, since many kinds of wireless communication systems such as wireless fidelity (Wi-Fi), worldwide interoperability for microwave access (WiMAX) or long-term evolution (LTE) (-Advanced) have turned into voracious consumers. Overcoming this shortfall is a critical issue in wireless communication. Drastic improvements of transmission rate and system capacity are required towards 5th generation mobile communications (5G) [1]. Promising solutions are to exploit millimeter wave bands for their rich spectrum resources and shrinking the cell size for improved area spectral efficiency [2,3]. The main problem with using the millimeter wave band is its link budget shortfall. The propagation loss is large and radio frequency (RF) components such as high power amplifiers (HPAs) have limited performance in the millimeter wave band. Application of massive multiple-input multiple-output (MIMO) [4,5,6,7] is one of the most promising palliatives. Massive MIMO can provide large beamforming gain with huge numbers of arrayed antenna elements without high-performance high-cost RF components [8]. Another approach to obtain higher capacity is multiuser MIMO, in which user equipments (UEs) are spatially multiplexed so that they can use the same frequency channel at the same time [9].

Channel environments in the millimeter wave band are considered to be dominated by the Line-of-Sight (LoS) component since the base station (BS) or UEs are required to have highly directive antennas to obtain sufficient transmission/reception gain. In this situation, multiuser diversity gain is expected to increase the system capacity [10] since the inter-user correlation between the UEs is lower than intra-user correlation. In other words, antenna elements in a UE are highly correlated to each other and this causes a large level gap between the 1st and 2nd eigenvalues in the LoS dominant single user MIMO channel. Therefore, higher eigenmodes cannot fully contribute to improving the multistream transmission efficiency. Meanwhile, to spatially multiplex several UEs, the BS requires channel state information at the transmitter (CSIT) to suppress inter-user interference (IUI). The accuracy of CSIT is degraded by the channel time variation created by the movement of the UEs or objects around the UEs. Inaccurate CSIT causes incomplete IUI suppression, which degrades the signal-to-interference power ratio (SIR) performance of multiuser MIMO [11]. We have verified one of the massive MIMO benefits, enhanced robustness of multiuser transmission in time-varying-channel environments [12]. Large beamforming gain is, thanks to the excess degrees of freedom (DoF), still higher than IUI leakage after user movement. This contributes to extend the CSI estimation period as well as reducing the feedback overhead [13].

However, critical issues remain with massive MIMO in the millimeter wave band; CSI per antenna between transmitter and receiver is noisy due to the short link budget as mentioned above. Moreover, the computation complexity imposed by eigenmode transmission is excessive due to singular value decomposition (SVD) calculation. This becomes especially serious in wideband systems. To realize 1st eigenmode reception at a UE with multiple antenna elements, the beam is simply steered toward the BS antenna array. To estimate angle of arrival (AoA) is the straightforward way and multiple signal classification (MUSIC) [14] or estimation of signal parameters via rotational invariant techniques (ESPRIT) [15] are well known legacy algorithms. They were originally developed for signal source analysis, hence auto-correlation matrix calculation with signal outputs via multiple antennas and SVD operation are essential. Their computation costs will be significant with a massive array. As the effective solutions, hybrid analog/digital beamforming approaches have been studied [8,16,17,18,19]. Analog beamforming can reduce costly RF chains and the computation costs associated with digital processing. It requires beam training or search with the use of pre-determined beam patterns, and so causes an overhead. Exploiting the LoS dominant (i.e., sparse) nature of millimeter wave communication, compressed sensing based simplified CSI estimation schemes has also been investigated [18]. Though these studies also address CSI for supporting multiple stream transmission, evaluations have been limited to the single user MIMO scenario. As indicated above, there remains the possibility of further enhancing the system capacity in millimeter wave channels via multiuser MIMO. From the implementations viewpoint, multiuser MIMO transmission with multistream per UE requires SVD operation and block diagonalization (BD). It causes difficulty in hardware implementation. The equipment, which requires high speed and large amount of computation, should be operated as simple as possible. Assuming that we assign single stream to each UE, such signal processing include CSI estimation can be significantly simplified even though full digital processing and it is expected to save the hardware resource with optimized design. System capacity can be enlarged by spatially multiplexing a number of UEs. Given the above features, we focus on multiuser massive MIMO with only 1st eigenmode transmission to each UE to achieve stable and high transmission capacity, even in high mobility situations.

This paper proposes an approximate beamforming scheme based on highly efficient CSI estimation. It is based on frequency domain CSI interpolation schemes using sparsely arranged training subcarriers, on which power density is concentrated. Although numerous interpolation schemes have been investigated [20,21], our proposal only extracts phase components with simple linear least squares regression, by using quite limited number of subcarriers. Furthermore, training subcarriers are dispersed on plural antenna elements to further reduce peak-to-average power ratio (PAPR) per antenna element. It reduces the burden of complexity and additive noise effect by roughly estimating the CSI from the LoS component. The two key contributions of this paper are: (1) the robustness of 1st eigenmode transmission is validated by comparisons of the achievable throughput with the parameter of the stream number allocated per UE; and (2) a simplified beamforming scheme based on highly efficient CSI estimation is introduced and verified.

The rest of this paper is organized as follows. Section 2 defines the system model and presents the methodology of multiuser massive MIMO eigenmode transmission. Section 3 describes the proposed scheme: simplified beamforming based on highly efficient CSI estimation. Computer simulation results are shown in Section 4. Finally, Section 5 concludes the paper. Throughout the paper, normal letters represent scalar quantities, bold lowercase letters indicate vectors and uppercase letters indicate matrices. |.|, ‖.‖, (.)*^T^*, and (.)*^H^* represent absolute values, Frobenius norm, transpose and conjugate transpose, respectively.

## 2. System Definition

### 2.1. System and Channel Model

**Figure 1:**
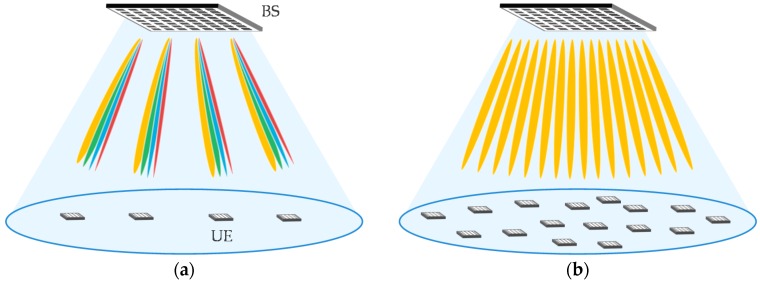
System model: (**a**) Multistream transmission per user equipment (UE): *Nu* = 4 and *Ns* = 4; and (**b**) 1st eigenmode transmission per UE: *Nu* = 16 and *Ns* = 1.

This paper examines the downlink transmission of a single cell multiuser massive MIMO system. BS with an *Nt* element uniform planar array (UPA) serves *Nu* UEs with *Nr* element UPA where each UE is assigned *Ns* signal streams. To ensure the LoS environment and reduce the probability of human blockage [22], BS is assumed to be located on the ceiling and UEs are facing straight up, as shown in Figure 1.

Assuming orthogonal frequency division multiplexing (OFDM) transmission, we define the channel matrix per subcarrier, **H** ∈ ℂ*^Nu^^Nr×Nt^*, as follows:

(1) $$H=\;{\left[\;\begin{array}{ccccc} H_{1}^{T} & \cdots & H_{i}^{T} & \cdots & H_{Nu}^{T}\; \end{array}\;\right]}^{\;T}$$

where **H***_i_* ∈ ℂ*^Nr×Nt^* denotes the channel sub matrix between the *i*-th UE and BS. Note these expressions are per subcarrier so indices are omitted. A Rician fading channel is considered so **H***_i_* is expressed using Rician *K*-factor as:

(2) $$H_{i}=\sqrt{\frac{K}{K+1}}\;H_{LoS,i}+\sqrt{\frac{1}{K+1}}\;H_{NLoS,i}\;$$

**H***_LoS,i_* is determined by the spatial relationship of the *i*-th UE and BS:

(3) $$H_{LoS,i}=\left[\begin{array}{ccc} e^{-j2\pi \frac{d_{11}}{\lambda }} & \cdots & e^{-j2\pi \frac{d_{1Nt}}{\lambda }}\\ \vdots & \ddots & \vdots \\ e^{-j2\pi \frac{d_{Nr1}}{\lambda }} & \cdots & e^{-j2\pi \frac{d_{NrNt}}{\lambda }} \end{array}\right]$$

where *d_mn_* is the distance between the *m*-th UE antenna element and the *n*-th BS antenna element. *λ* is the carrier wavelength. Path loss component is omitted here. The channel time variation of **H***_LoS,i_* is simulated by the spatial relationships between the UEs and the BSs determined by UE movement. **H***_NLoS,i_* is the non-line-of-sight (NLoS) component from the scatters, which are uniformly sited around the UEs. To consider the spatial correlation between BS antenna elements, independent identically distributed (i.i.d.) Rayleigh fading channels are converted into correlated channels using the Kronecker model [23].

(4) $$H_{NLoS,i}=R_{rx,i}^{1/2}\;H_{iid,i}\;{\left(R_{tx,i}^{1/2}\right)}^{T}$$

where correlation matrices **R***_tx,i_* ∈ ℂ*^Nt×Nt^* and **R***_rx,i_* ∈ ℂ*^Nr×Nr^* are expressed as,

(5) $$R_{tx,i}=\left(\begin{array}{cccc} 1 & \rho_{tx1,tx2}^{\left(i\right)} & \cdots & \rho_{tx1,txNt}^{\left(i\right)}\\ \rho_{tx2,tx1}^{\left(i\right)} & 1 & \cdots & \rho_{tx2,txNt}^{\left(i\right)}\\ \vdots & \vdots & \ddots & \vdots \\ \rho_{txNt,tx1}^{\left(i\right)} & \rho_{txNt,tx2}^{\left(i\right)} & \cdots & 1 \end{array}\right)$$

(6) $$R_{rx,i}=\left(\begin{array}{cccc} 1 & \rho_{rx1,rx2}^{\left(i\right)} & \cdots & \rho_{rx1,rxNr}^{\left(i\right)}\\ \rho_{rx2,rx1}^{\left(i\right)} & 1 & \cdots & \rho_{rx2,rxNr}^{\left(i\right)}\\ \vdots & \vdots & \ddots & \vdots \\ \rho_{rxNr,rx1}^{\left(i\right)} & \rho_{rxNr,rx2}^{\left(i\right)} & \cdots & 1 \end{array}\right)$$

Spatial correlation coefficient between the *p-*th and the *q*-th antenna elements is derived as [24]:

(7) $$\rho_{p,q}=\frac{\int_{o}^{\pi }\int_{-\pi }^{\pi }A_{p}(\psi ,\theta )A_{q}(\psi ,\theta )\;G(\psi ,\theta )\;e^{-jW_{p.q}(\psi ,\theta )}d\psi d\theta }{\int_{o}^{\pi }\int_{-\pi }^{\pi }A_{p}(\psi ,\theta )A_{q}(\psi ,\theta )\;G(\psi ,\theta )\;d\psi d\theta }$$

UPA is arranged on *xy* plane, *ψ* [rad] is the AoA or angle of departure (AoD) from the *x-*axis toward the positive *z*-axis, and *θ* [rad] is the angle from the positive *y*-axis toward the *xz* plane. *A_p_*(*ψ*,*θ*) [dB] is radiation pattern of the *p*-th antenna element. Assuming that from 3GPP 3D channel model [25], it is defined as follows:

(8) A(ψ,θ)=−min[−{A(ψ)+A(θ)}, 30]

(9) $$A(\psi )=-\min\left[12\;{\left(\frac{\psi -\pi /4}{\psi_{3\text{dB}}}\right)}^{2},\;30\right]$$

(10) $$A(\theta )=-\min\left[12\;{\left(\frac{\theta -\pi /4}{\theta_{3\text{dB}}}\right)}^{2},\;30\right]$$

where *ψ*_3dB_ and *θ*_3dB_ denote half power beamwidth (HPBW), and are set to 1.135 (=65°) [25]. The above settings are commonly applied to the transmitter and receiver side. *G*(*ψ*,*θ*) is the joint probability density function of AoAs, which is assumed to follow Laplacian distribution [26]:

(11) $$G(\psi ,\theta )=\frac{1}{\sqrt{2}\sigma_{\psi }}e^{-\left|\frac{\sqrt{2}\psi }{\sigma_{\psi }}\right|}\;\frac{1}{\sqrt{2}\sigma_{\theta }}e^{-\left|\frac{\sqrt{2}\theta }{\sigma_{\theta }}\right|}$$

Standard deviation value, *σ_ψ_* and *σ_θ_*, are set to 0.087 (=5°) to simulate the LoS dominant channel. *W_p.q_*(*ψ*,*θ*) is phase difference between the *p*-th and the *q*-th antenna elements, where suffixes *x* and *y* indicate row-wise and column-wise directions, respectively.

(12) $$W_{p,q}(\psi ,\theta )=2\pi \frac{\delta x(p_{x}-q_{x})}{\lambda }\cos\psi \sin\theta +2\pi \frac{\delta y(p_{y}-q_{y})}{\lambda }\cos\theta$$

Since the calculation of Equation (7) for massive antenna elements requires heavy complexity, integrations are approximated using clustered angular spread [25] (Table 7.3-3). Time variation of the NLoS component follows Jakes’model [27].

### 2.2. MU-MIMO Eigenmode Transmittion

When the number of UE antenna elements is larger than that of the transmission stream, i.e., *Nr* > *Ns*, BS performs space division multiplexing (SDM) or beamforming in beam space [28]. The *i*-th UE obtains channel matrix **H***_i_* and computes the SVD.

(13) $$H_{i}=[U_{i}\;{\bar{U}}_{i}]\Sigma_{i}V_{i}^{H}$$

where **U***_i_* ∈ ℂ*^Nr×Ns^*, **Σ***_i_* ∈ ℂ*^Nr×Nt^*, and **V***_i_* ∈ ℂ*^Nt×Nt^* represent left singular matrix, singular value matrix whose diagonal elements are arranged in descending order, and right singular matrix, respectively. Using **U***_i_^H^* for the *i*-th UE provides effective MIMO channel matrix for BS, **H**’ ∈ ℂ*^NuNs×Nt^*;

(14) $$H^{\prime }=\left[\;\begin{array}{ccccc} {\left(U_{1}^{H}H_{1}\right)}^{T} & \cdots & {\left(U_{i}^{H}H_{i}\right)}^{T} & \cdots & (U_{Nu}^{H}H_{Nu} \end{array}{)}^{T}\right]{\;}^{T}$$

**Figure 2:**
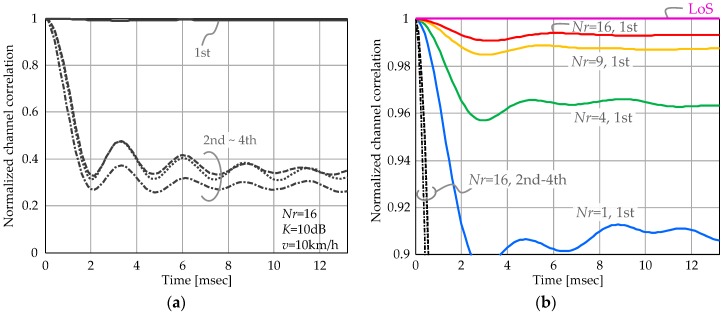
Channel time variation: (**a**) channel correlation fluctuation of four eigenmodes when *Nr* = 16; and (**b**) channel correlation of the 1st eigenmode with increased number of UE antenna elements, *Nr*.

BS calculates the precoding weight via BD [28]. Here, the transmission/reception weight for the 1st eigenmode steers the beam so as to obtain large gain. It extracts the path for the stable LoS component and, by comparison, suppresses NLoS components that have extremely strong time variation characteristics. Since massive MIMO enhances beamforming efficacy, it can be expected to further improve system robustness to the time varying channels. This fundamental characteristic is verified here. Figure 2 shows the normalized correlation of the effective channel vector defined as:

(15) $$C_{j}(t)=\;\frac{\left|\left\{\;u_{ij}^{H}H_{i}(0)\right\}\;{\left\{\;u_{ij}^{H}H_{i}(t)\right\}}^{H}\right|}{\left\|\;u_{ij}^{H}H_{i}(0)\right\|\;\left\|\;u_{ij}^{H}H_{i}(t)\right\|}\;$$

where **H***_i_*(*t*) represents the channel matrix for the *i*-th UE at instant *t*; it is composed of the channel coefficient between *Nr* UE antenna and *Nt*(=256) BS antenna elements. **u***_ij_* ∈ ℂ*^Nr×^*^1^ is singular vector for the *j*-th eigenmode, i.e., **U***_i_* = [**u***_i_*_1_,...,**u***_ij_*,...,**u***_iNs_*]. Note that **U***_i_* is obtained from the CSI at *t* = 0. UE speed is assumed to be 10 km/h and Rician *K* factor is 10 dB. Figure 2a plots channel correlation fluctuation of four eigenmodes when *Nr* = 16. As shown, the correlations of the 2nd–4th eigenmodes rapidly decrease and fluctuate at a lower value while that of the 1st eigenmode is almost always 1. Multistream transmission per UE is considered to be severely affected by the channel time variation, especially in the 2nd and higher order eigenmodes. Figure 2b depicts channel correlation of the 1st eigenmode over time with the number of UE antenna elements, *Nr*, as the parameter. Increasing *Nr* can suppress the channel fluctuation impact. These fundamental results yield the expectation that 1st eigenmode transmission with large antenna arrays can achieve quite stable multiuser SDM performance.

## 3. Simplified Beamforming by High Efficient CSI Estimation

As discussed in the previous section, the 1st eigenmode is mostly formed by the LoS component (NLoS components are suppressed). Two key challenges are reviewed here; CSI estimation in the short link budget situation and computation complexity for SVD, which is required for each subcarrier. This section presents a simplified beamforming scheme by approximately extracting CSI from the LoS component in order to alleviate the calculation cost as well as the additive noise effect.

**Figure 3:**
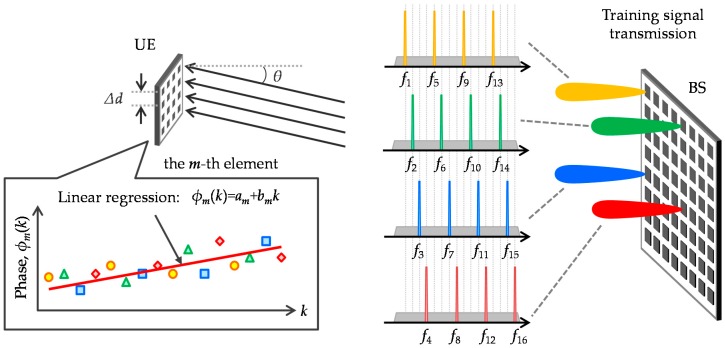
Proposed high efficient channel state information (CSI) estimation.

The proposed scheme is illustrated in Figure 3. First, a training signal is sent by a few antenna elements and subcarriers, e.g., 16 of the 256 elements at equal intervals and four of the 2048 subcarriers per antenna element without overlap. When total transmission power is constant, power density is concentrated on the limited number of subcarriers. Signal-to-noise power ratio (SNR) for the selected subcarriers is improved by 10log_10_(2048/4/16) = 15.1 dB before beamforming. In addition, reducing the number of subcarriers can suppress PAPR per antenna element, which alleviates the need for input backoff (IBO) for training signal transmission. Second, each UE antenna element receives the training signals as if they had been transmitted from a single antenna element. UE then obtains CSI for received subcarriers using known training sequence and then calculates relative CSI, which represents the relationship of the CSI between the reference antenna element and the CSI of the other ones. Next, the phase component of the relative CSI is extracted. Third, phase information for null subcarriers is interpolated by linear regression. Let *c*, *λ*, *f_c_* (=*c*/*λ*), *f_s_* and *d_m_* be the light speed, wavelength, carrier frequency, subcarrier spacing, and distance between the *m*-th UE antenna element and the BS, respectively; relative phase component of LoS channel at the *k*-th subcarrier, exp{*jφ_m_*(*k*)}, is expressed as,

(16) $$\begin{array}{lll} e^{j\phi_{m}(k)} & = & e^{-j2\pi \frac{f_{c}+kf_{s}}{c}(d_{m}-d_{1})}\\ & = & e^{-j2\pi \frac{f_{c}}{c}\Delta d}\cdot e^{-j2\pi \frac{kf_{s}}{c}\Delta d} \end{array}$$

where *Δd* = *d_m_* − *d*_1_. Here, phase fluctuation in the frequency domain is due to the term exp{−*j*2*π*(*kf_s_*/*c*)*Δd*}. In millimeter wave communication, inter-element space becomes much smaller than BS-UE distance so the relative phase information can be expressed as an array factor. Assuming a linear array with half wavelength spacing, *Δd* = (*λ*/2)(*m* − 1)sin*θ* where *θ* is AoA. From this we can derive:

(17) $$\begin{array}{lll} e^{j\phi_{m}(k)} & = & e^{-j2\pi \frac{1}{2}(m-1)\sin\theta }\cdot e^{-j2\pi \frac{kf_{s}}{c}\frac{\lambda }{2}(m-1)\sin\theta }\\ & = & e^{-j\pi (m-1)\sin\theta }\cdot e^{-j\pi \frac{kf_{s}}{B}\frac{B}{f_{c}}(m-1)\sin\theta } \end{array}$$

where *B* is the channel bandwidth. It is obvious that −1/2 ≤ (*kf_s_*/*B*) ≤ 1/2. When the system parameters are set to *f_c_* = 20 GHz and *B* = 400 MHz, fraction bandwidth, *B*/*f_c_*, is 0.02. We consider that *m* = 16 and *θ* = 45° at the limit, so in-band phase fluctuation is around 10% of 360°. Since this condition can be satisfied, linear regression can precisely extract the LoS component using extremely reduced training subcarriers. Relationship between *ϕ_m_*(*k*) and *k* can be described as:

(18) $$\phi_{m}(k)=a_{m}+b_{m}k$$

*a_m_* and *b_m_* can be obtained by the well-known least squares method (LSM) as follows:

(19) $$\left[\begin{array}{c} a_{m}\\ b_{m} \end{array}\right]={\left(X^{H}X\right)}^{-1}X^{H}Y$$

where

(20) $$X=\left[\begin{array}{cc} 1 & \vdots \\ 1 & k^{\prime }\\ 1 & \vdots \end{array}\right],\;Y=\left[\begin{array}{c} \vdots \\ \phi_{m}(k^{\prime })\\ \vdots \end{array}\right]$$

*k’* represents the subcarrier indices used for the training signal. Above operation is performed through the *m*-th element. Fourth, reception weight vector for the *i*-th UE is obtained as **U***_i_* = ***ϕ****_i_* = [*ϕ*_1_(*k*)*,...,ϕ_Nu_*(*k*)]*^T^*. Assuming time division duplex (TDD), UE should calibrate CSI for transmission beamforming since uplink and downlink signals go through different circuits, e.g., HPA and low noise amplifier (LNA). CSI for transmission can be obtained by applying a reciprocity calibration coefficient [29,30]. Last, UE transmits training signal via beamforming and BS estimates CSIT to calculate MU-MIMO precoding weight. Here, BS can use an existing CSI estimation scheme since the link budget is improved by the beamforming gain at the UE side. As a result, BS can obtain effective MIMO channel matrix, **H**’, as shown in Equation (14). If necessary, the highly efficient CSI estimation shown in Figure 3 can also be applied to the uplink where each UE uses different subcarrier/antenna combination without overlap.

Table 1 summarizes the computation complexities defined as the required number of complex multiplications. Complexity of the proposed scheme is derived from Equation (19), where *Nc* and *Np* indicate the subcarrier number for signal transmission and for training signal transmission in the proposed scheme, respectively. That of SVD is based on QR-decomposition and Householder reflections [31].

## 4. Results and Discussion

### 4.1. System Level Simulation

Principal simulation parameters are listed in Table 2. BS and UEs use UPAs with 256 and 16 elements, respectively. BS height is assumed to be 30 m and UEs with 1.5 m height are uniformly distributed in the single cell with radius of 20 m, as shown in Figure 1. SNR, given for the antenna elements linking BS and UE, is assumed to be 10 dB. Assuming a Rician fading channel with *K* = 10 dB, the multipath component is modeled as 11 path exponential decay with 3 dB attenuation for each 10 ns as per [32]. Spatial correlation, i.e., **R***_tx_*_,*i*_, **R***_rx_*_,*i*_, and LoS channel, change with UE rotation on the horizontal plane. CSI is updated every 1.3 ms. This corresponds to 200 symbols, each with 6.67 μs symbol duration. CSI estimation error due to the receiver noise is excluded in order to evaluate the impact of the outdated CSI. We compare the following three schemes:

- Case 1: Four stream per UE via BD;
- Case 2: One stream per UE via BD (1st Eigenmode); and
- Case 3: One stream per UE via simplified beamforming (proposal).

In Case 1, UE is assumed to perform minimum mean square error (MMSE) detection at every symbol reception. Cases 2 and 3 simply steer the beam to the BS array by using CSI at the update instance. We evaluate achievable signal-to-interference plus noise power ratio (SINR) and throughput performance. Throughput is determined based on modulation coding scheme (MCS) matches as the received SINR [33,34]. The relationship is defined in Table 3. Since the original relationship was defined for an additive white Gaussian noise (AWGN) channel, we added a margin of 6 dB to ensure a conservative evaluation. User scheduling effect is taken into account by separating the UEs by least 3 m.

### 4.2. Simulation Results

#### 4.2.1. Performance of First Eigenmode Transmission

**Figure 4:**
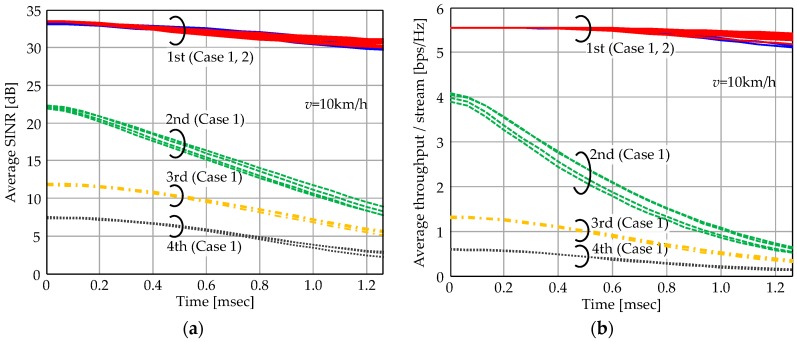
Time variant characteristics within the CSI estimation period. Case 1: *Nu* = 4, *Ns* = 4; Case 2: *Nu* = 16, *Ns* = 1. (**a**) Average SINR per stream versus elapsed time. (**b**) Average throughput per stream versus elapsed time.

First we observe time variant characteristics within the CSI estimation period. Figure 4a shows average SINR fluctuation of each stream and corresponding throughput performance is shown in Figure 4b. The average values plotted include all subcarriers with various UE distributions. Here we compare Cases 1 and 2. Total transmission stream is set to *NuNs* = 16; 4 UEs with four streams for Case 1 and 16 UEs with single stream for Case 2. Maximum beamforming gain is calculated as 10log_10_{*NtNr*/(*NuNs*)} = 10log_10_(256 × 16/16) = 24 dB and the 1st eigenmode properly yields these gain values in Cases 1 and 2. However, higher eigenmodes for Case 1 exhibit lower SINR than the 1st one and they rapidly decrease as time progresses. Their achievable throughput values are lower than 1 bps/Hz at *t* = 1.3 ms. As Case 2 allocates a single stream per UE, SINR values of all signal streams remain high so the throughput degradation is minimal.

Note the achievable throughput values are directly converted from SINR in the evaluation. Practical MCS need to be determined with some margin considering the unpredictable SINR degradation possible with the time varying channel. If appropriate MCS cannot be selected, expected throughput cannot be achievable. The 1st eigenmode approach can suppress such MCS selection error since it realizes stable SINR performance. Therefore, the superiority of the 1st eigenmode transmission of Case 2 is expected to be demonstrated more clearly in actual use.

#### 4.2.2. Performance of Simplified Beamforming

**Figure 5:**
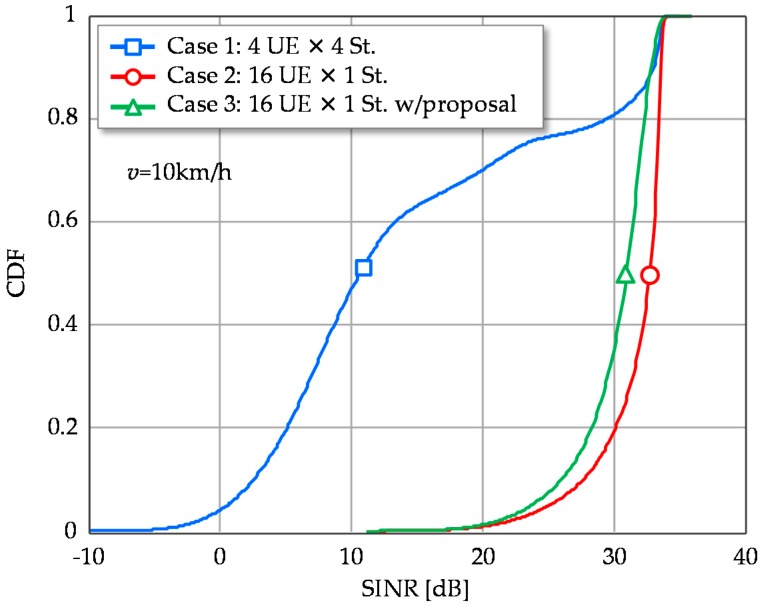
Cumulative distribution functions (CDFs) of SINR per signal stream.

**Figure 6:**
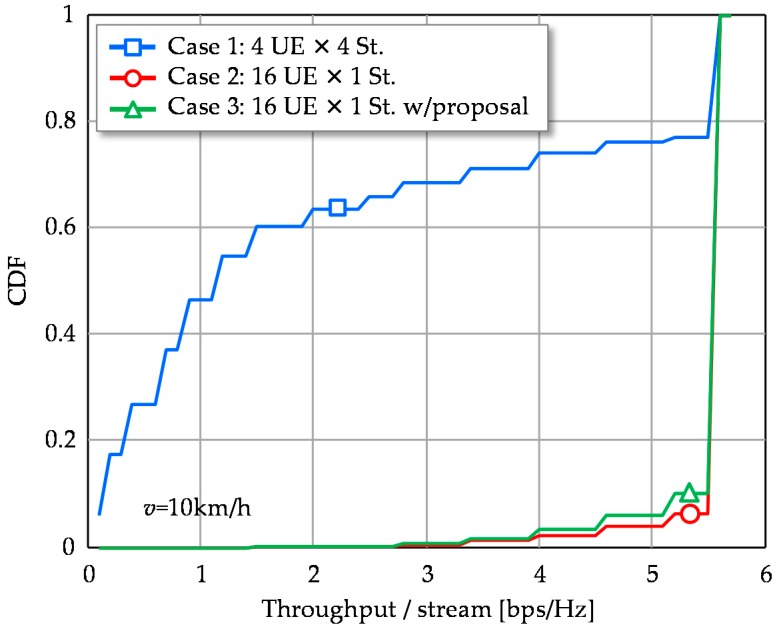
CDFs of throughput per signal stream.

The following examines the proposed beamforming scheme in Case 3. Figure 5 shows the cumulative distribution functions (CDFs) of SINR per stream in a CSI estimation period. Case 2 exhibits high stable SINR distribution compared to Case 1 as its 2nd, 3rd and 4th eigenmodes cause relatively low SINR. The proposed scheme of Case 3 can match the SINR performance of Case 2. Though the proposed scheme is based on the simplified approximate approach of 1st eigenmode transmission, its degradation at CDF = 50% is only about 3 dB. It is still superior to Case 1 and achieves large SINR, more than 25 dB with 90% probability, where maximum MCS is available. Therefore, the 3 dB SINR degradation of the proposed scheme is negligible in terms of throughput, as shown in Figure 6. Throughput performance of multistream transmission (Case 1) is largely degraded due to the instability of the higher eigenmodes.

**Figure 7:**
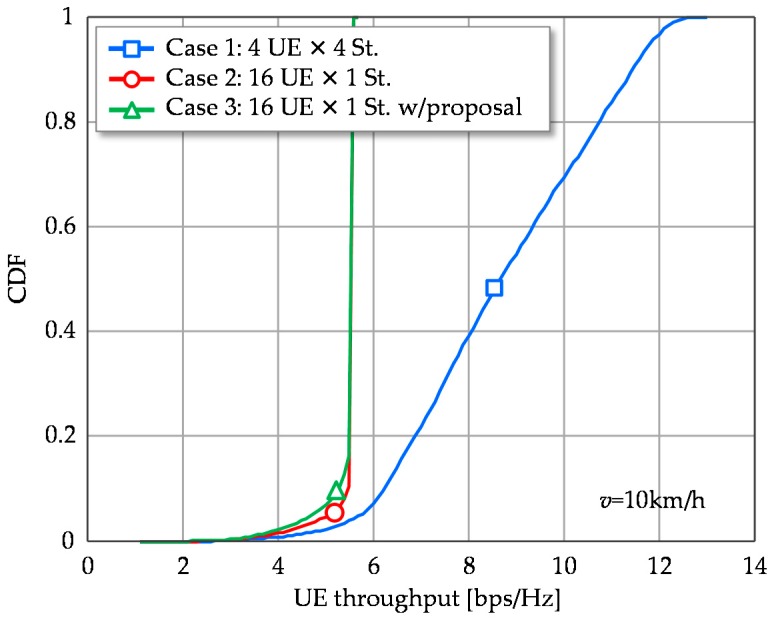
CDFs of UE throughput.

Figure 7 shows the CDFs of UE throughput. Case 1, with multistream transmission, outperforms the other single stream transmission schemes. Though it potentially deserves to provide a higher transmission rate per UE, it sacrifices system throughput and is strongly impacted by the channel time variation.

**Figure 8:**
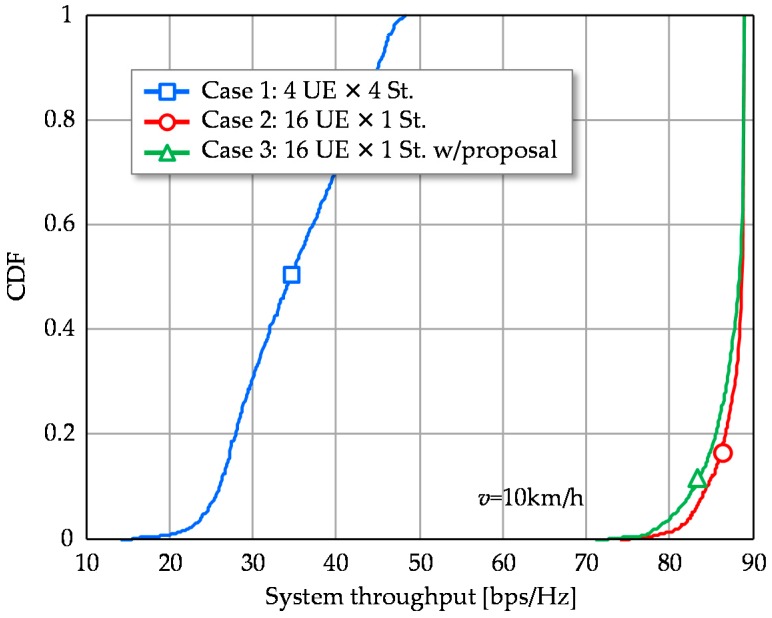
CDFs of System throughput.

Figure 8 shows the CDFs of system throughput performance. As shown, Cases 2 and 3 provide quite large system throughput compared to Case 1. Under the constraint of total transmission stream number, multiplexing a large number of UEs to use a single stable stream is effective from the system throughput viewpoint.

**Figure 9:**
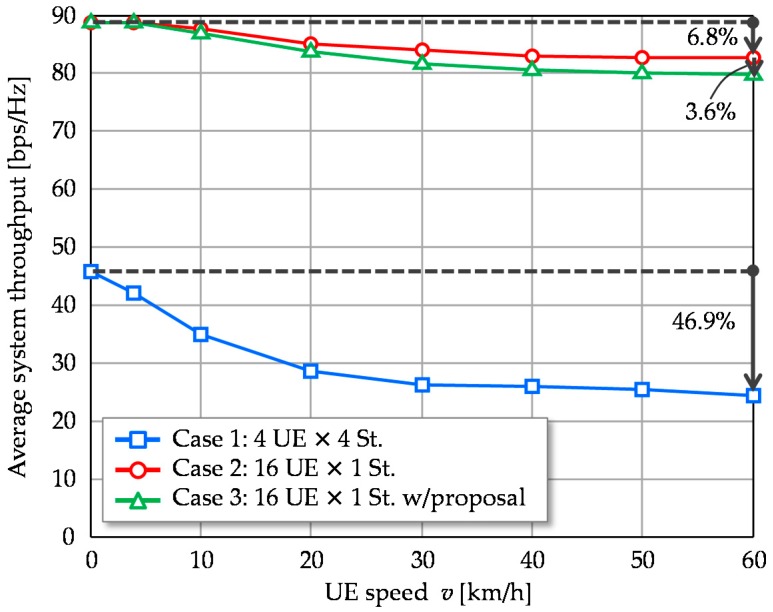
Average system throughput versus UE speed.

Figure 9 plots the average system throughput versus UE speed, *v*. With Case 1, system throughput is decreased as movement speed increases and is reduced by 46.9% at 60 km/h. The reductions observed in Cases 2 and 3 are 6.8% and 10.4%, respectively. The gap between them is only 3.6%. Multiuser MIMO with 1st eigenmode transmission as well as our proposal can handle such high mobility situations, the movement speed is expected to be around 10 km/h considering the small cell use cases. It should be noted that extending the CSI feedback period *M* times corresponds to increasing the UE speed *M* times. Therefore, this evaluation is useful in understanding the impact of long CSI feedback periods from the viewpoint of control overhead savings.

**Figure 10:**
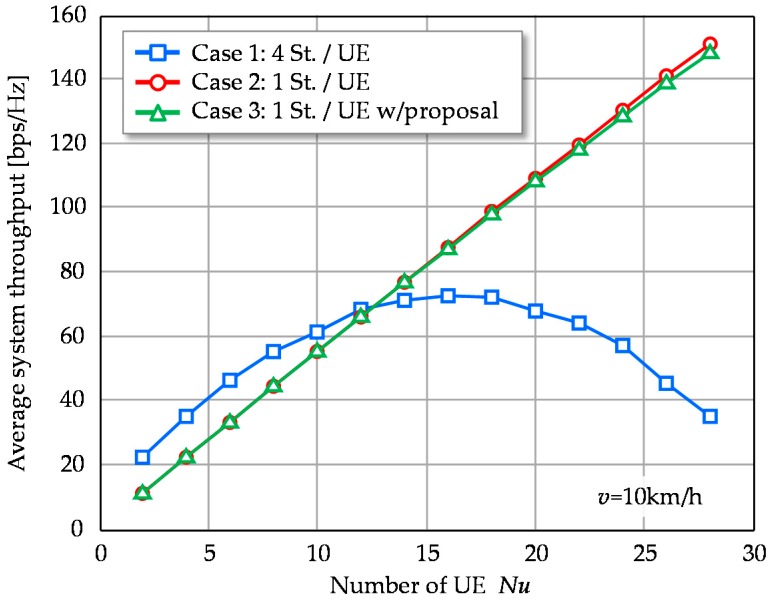
Average system throughput versus number of multiplexed UEs.

Finally, average system throughput in terms of multiplexed UE number, *Nu*, is evaluated and the result is shown in Figure 10. Case 1 achieves its peak system throughput at *Nu* = 16. In addition, Case 1 provides higher system throughput than Cases 2 and 3 at *Nu* < 12. This is because the total number of signal streams for Case 1 is four times those of Cases 2 and 3 under the same spatially multiplexed UE number. However, it devours the DoF resources and results in inefficient UE throughput. The system throughput finally decreases as *Nu* increases further. Meanwhile, the system throughput of Cases 2 and 3 linearly increases with *Nu* in the plotted region and they outperform Case 1 when *Nu* > 14. There remain enough DoF resources with single stream transmission per UE and all UEs exploit the 1st eigenmode, which provides the highest gain. Since UEs are spatially de-correlated, 1st eigenmodes can keep their high gain and thus higher spectral efficiency is achieved even in large number of *Nu*.

Above results prove that exploiting the 1st eigenmode can realize stable multiuser MIMO transmission in time varying channels. System throughput can be significantly enhanced by spatially multiplexing a large number of UEs. Furthermore, we have confirmed the effectiveness of the proposed beamforming scheme based on simplified and high efficient CSI estimation. From the complexity estimate in Table 1 and the parameters used in Table 2, the conventional SVD and the proposed scheme require 278,528,000 and 20,480,000 multiplications, respectively; thus a 92.6% complexity reduction can be achieved. To emphasize this advantage, we examine the compressed sensing based CSI estimation scheme in [18], referred as multi-grid orthogonal matching pursuit (MG-OMP). When *L,* *N*_T_^Beam^, *N*_R_^Beam^, and (*G*_0_,*G*_1_) be the number of scatters, transmission/reception training beams, and discrete angle sets, respectively, its complexity is estimated to be *O*(*LN*_T_^Beam^*N*_R_^Beam^(*G*_0_^2^ + *G*_1_^2^)), i.e., 37,365,760 if we set *L* = 10, *N*_T_^Beam^ = *N*_R_^Beam^ = 32, (*G*_0_,*G*_1_) = (60,7) as the derived parameters. The proposed scheme offers a 45.2% reduction in complexity. However, it should be understood that our proposal can ease hardware implementation since it does only require multiplications with matrices and a vector with simplified algorithms. This is one of the remarkable advantages of our proposal.

### 4.3. Discussion

**Figure 11:**
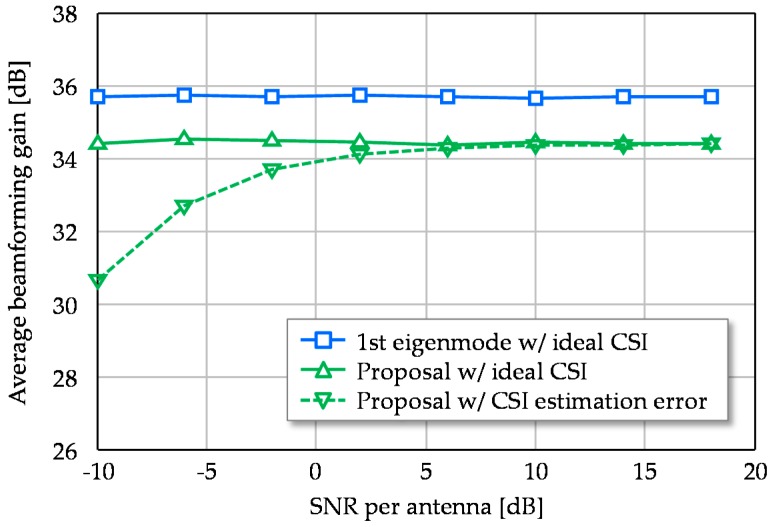
Average beamforming gain versus signal-to-noise power ratio (SNR).

The above simulations assumed a fixed SNR (=10 dB) situation to permit a comprehensive evaluation. When we assume a total transmission power of 10 dBm, 0 dBi antenna gain, −174 dBm/Hz noise density and free space propagation, average reception SNR in the SISO case is about 9 dB in the simulation environment. Of course, the actual value depends on system parameters and deployment environment and the received signal strength significantly impacts the capability of the proposed CSI estimation scheme. To discuss the application region, Figure 11 plots average beamforming gain versus SNR where 1st eigenmode transmission is compared to the proposed scheme. It additionally plots the result of the proposed scheme with CSI estimation error due to additive noise. Under the total transmission power constraint, reducing the training subcarriers from 2048 to four strengthens the transmission power density per subcarrier, per antenna element, by 10log_10_(2048/4/16) = 15.1 dB. In addition, thinning out the training subcarriers also reduces PAPR and thus the transmission power can be raised thanks to easing IBO; this reduction is assumed to be 6 dB or so [35]. As seen by the results, even with CSI estimation error, the proposed scheme retains comparable beamforming gain to the ideal CSI case in the low SNR region around 0 dB. Though the practical gain 15.1 + 6 = 21.1 dB is much smaller than the beamforming gain 36 dB, the lack of the gain does not cause serious degradation on our proposed scheme, even in low SNR region. This validates the throughput performance evaluations in Section 4.2, which assumed ideal CSI.

As a result, our proposal can eliminate the link budget shortfall for CSI estimation in the millimeter waveband. It should be noted that assuming OFDM, timing extraction for fast Fourier transform (FFT) windowing for the CSI estimation on the reduced subcarrier is a critical issue since it is performed in the time domain, i.e., the gain provided by the reduced number of training subcarriers cannot be obtained. Meanwhile, millimeter wave small cells can be supported by control signaling from macro cell using micro wave band [36]. It will be possible to know the FFT windowing timing by synchronizing the macro cell system.

## 5. Conclusions

This paper verified the effectiveness of 1st eigenmode transmission in multiuser massive MIMO assuming LoS dominant channels in the millimeter wave band. Because of the 1st eigenmode’s robustness and high-gain property, quite a large number of UEs can be spatially multiplexed; it is effective in enhancing the system throughput rather than UE throughput. Additionally, we proposed simplified beamforming with highly efficient CSI estimation in order to ease heavy computation complexity imposed by 1st eigenmode transmission. It can also suppress the additive noise effect and thus improve CSI estimation accuracy. Our approximate approach achieved performance comparable to that of rigorously applied 1st eigenmode transmission.

## Figures and Tables

**Table 1:** Computation complexity.

Precoding Scheme	Complexity
Proposed scheme	10*NcNpNr*
SVD	2*Nc*(*NtNr*^2^ + *Nr*^3^)

**Table 2:** Simulation parameters.

Parameters	Values
Carrier frequency	20 GHz
Bandwidth	400 MHz
Number of FFT points	2048
Number of subcarriers; *Nc*	2000
Number of subcarriers for proposed CSI estimation; *Np*	64 (4 subcarriers × 16 antennas)
Number of BS antennas; *Nt*	256 (16 × 16) UPA, 0.5*λ* spacing, HPBW = 65°
Number of UE antennas; *Nr*	16 (4 × 4) UPA, 0.5*λ* spacing, HPBW = 65°
Number of UEs; *Nu*	Case 1: 4; Cases 2, 3: 16
Tx streams per UE; *Ns*	Case 1: 4; Cases 2, 3: 1
SNR	10 dB @ SISO
Channel model	Rician fading, *K* = 10 dB 11 path exponential decay RMS delay spread: 14 ns
Tx/Rx Angular spread	5°/5°
Precoding	BD/Eigenmode transmission
Postcoding	MMSE
Symbol duration	6.67 μs
CSI estimation period	1.334 ms (200 symbol)
UE speed; *v*	10 km/h (*f_D_T_S_* = 1.2 × 10^−3^)

**Table 3:** Achievable throughput for signal-to-interference plus noise power ratio (SINR).

MCS Index	SINR (dB)	Throughput (bps/Hz)
0	-	0
1	1.00	0.1523
2	3.21	0.2344
3	5.43	0.3770
4	6.36	0.6016
5	8.14	0.8770
6	9.93	1.1758
7	11.71	1.4766
8	13.50	1.9141
9	15.29	2.4063
10	17.07	2.7305
11	18.86	3.3223
12	20.64	3.9023
13	22.43	4.5234
14	24.21	5.1152
15	26.00	5.5547
